# Anticipated regret in shared decision-making: a randomized experimental study

**DOI:** 10.1186/s13741-016-0031-6

**Published:** 2016-03-02

**Authors:** Rebecca M. Speck, Mark D. Neuman, Kimberly S. Resnick, Barbara A. Mellers, Lee A. Fleisher

**Affiliations:** Department of Anesthesiology and Critical Care, Center for Pharmacoepidemiology Research and Training, University of Pennsylvania, 3400 Spruce Street, Dulles 6, Philadelphia, PA 19104 USA; Evidera, 1417 4th Ave., Suite 510, Seattle, WA 98101 USA; Department of Anesthesiology and Critical Care, Leonard Davis Institute, University of Pennsylvania, 3400 Spruce Street, Dulles 6, Philadelphia, PA 19104 USA; Department of Psychiatry, University of Pennsylvania, 3535 Market St, Philadelphia, PA 19104 USA; Department of Psychology, Department of Marketing, University of Pennsylvania, 3720 Walnut St, Philadelphia, PA 19104 USA; Department of Anesthesiology and Critical Care, Center for Pharmacoepidemiology Research and Training, Leonard Davis Institute, University of Pennsylvania, 3400 Spruce Street, Dulles 6, Philadelphia, PA 19104 USA

**Keywords:** Breast cancer, Anticipated regret, Decision-making, Surgery

## Abstract

**Background:**

Explicit consideration of anticipated regret is not part of the standard shared decision-making protocols. This pilot study aimed to compare decisions about a hypothetical surgery for breast cancer and examined whether regret is a consideration in treatment decisions.

**Methods:**

In this randomized experimental study, 184 healthy female volunteers were randomized to receive a standard decision aid (control) or one with information on post-surgical regret (experimental). The main outcome measures were the proportion of subjects choosing lumpectomy vs. mastectomy and the proportion reporting that regret played a role in the decision made. We hypothesized that a greater proportion of the experimental group (regret-incorporated decision aid) would make a surgical treatment preference that favored the less regret-inducing option and that they would be more likely to consider regret in their decision-making process as compared to the control group.

**Results:**

A significantly greater proportion of the experimental group subjects reported regret played a role in their decision-making process compared to the control counterparts (78 vs. 65 %; *p* = 0.039). Recipients of the regret-incorporated experimental decision aid had a threefold increased odds of choosing the less regret-inducing surgery (OR = 2.97; 95 % CI = 1.25, 7.09; *p* value = 0.014).

**Conclusions:**

In this hypothetical context, the incorporation of regret in a decision aid for preference-sensitive surgery impacted decision-making. This finding suggests that keying in on anticipated regret may be an important element of shared decision-making strategies. Our results make a strong argument for applying this design and pursuing further research in a surgical patient population.

**Trial registration:**

Clinicaltrials.gov, NCT02563808.

**Electronic supplementary material:**

The online version of this article (doi:10.1186/s13741-016-0031-6) contains supplementary material, which is available to authorized users.

## Background

In recent years, the US health-care system has been redefining itself as one that strives to be patient-centered. Central to this idea is the belief that patients ought to be actively engaged in their health care (Barry & Edgman-Levitan 2012). One approach to accomplishing this goal is the use of “shared decision-making,” in which physician and patient share information in an attempt to improve the extent to which patients select treatments that match their underlying preferences (Wennberg et al. 2002). It is thought that shared decision-making can improve care and reduce costs and is encouraged and supported by the Affordable Care Act (ACA) (Oshima & Emanuel 2013).

Shared decision-making may be particularly important in clinical situations in which there is clinical equipoise, i.e., there is no best treatment paradigm. In these “preference-sensitive” conditions, the treatment choice depends on the unique values of the patient (Wennberg 2002). Although the use of decision aids has been shown to increase patient activation, improve knowledge, and aid in the realistic perception of outcome, the size of the effect varies across studies (Wennberg et al. 2010; Stacey et al. 2011). Furthermore, randomized control trials comparing the impact of decision aids with “no intervention,” “usual care,” or “alternate intervention” have found current decision aids to be no better than comparisons in improving satisfaction with decision-making, anxiety, health outcomes, or decisional regret (O’Connor et al. 2009; Goel et al. 2001).

In the current model of shared decision-making, patients are asked to make surgical decisions in which the risks of different outcomes are discussed and presented numerically. However, multiple studies have documented the public’s lack of the numerical skills essential to making informed medical decisions (Reyna et al. 2009). In the area of surgical treatment of breast cancer, external forces such as celebrity treatment choice rather than evidence can influence the desire for more aggressive treatment. Nancy Reagan’s choice of mastectomy over lumpectomy in the late 1980s (Lewin 1988; Nattinger et al. 1998) and more recently Angelina Jolie’s decision to have a prophylactic double mastectomy are two examples (Kamenova et al. 2014).

Recent psychological research has highlighted the role of anticipated regret—the fear of “buyer’s remorse”—as an important influence on individual choices across a range of domains, including health care (Zeelenberg 1999; Simonson 1992; Nelissen et al. 2011). While largely absent from past research on the design of decision aids, insights from research on anticipated regret may hold promise as a potential means of improving decision-making strategies.

In this study, we aimed to investigate whether surgical treatment choices for breast cancer would differ based on the decision aid the subjects were given. We hypothesized that subjects given an experimental decision aid which incorporated information about regret would be more likely to make a surgical treatment preference that was less regret-inducing and that they would be more likely to consider regret in their decision-making process as compared to subjects in the control group.

## Methods

We conducted a randomized experimental pilot study at the University of Pennsylvania among healthy female volunteers receiving decision aids for the surgical treatment of early-stage breast cancer. Early-stage breast cancer was chosen because although it has more than one treatment option available, there is little medical evidence to suggest one treatment option is superior to another. Consequently, the ultimate treatment choice is informed by patient preference and greatly influenced by shared decision-making strategies. Furthermore, early-stage breast cancer can be treated with increasingly invasive procedures, the more invasive of which has been associated with high rates of regret after surgery.

We incorporated information about the rate at which regret occurs after surgery into standard decision aids for breast cancer and assigned volunteers to receive the standard or regret-incorporated versions.

### Study participants and materials

The study received International Review Board approval through the University of Pennsylvania. A waiver for written informed consent was approved. Study subjects were healthy female volunteers, recruited through campus advertising, and offered a participant compensation of US$5. Subject recruitment and participation occurred in April 2013. There were no specific inclusion or exclusion criteria.

Study participants received decision aids on surgery for early-stage breast cancer (Additional files 1 and 2). We created two versions of the decision aid: one based on standard information and one that incorporated additional information on the rates of regret after surgical treatment. To develop the standard version, we relied on existing decision aids developed by the Informed Medical Decisions Foundation and selected those with the highest scores for the content, development process, and effectiveness criteria proposed by the International Patient Decision Aids Standards (IPDAS) collaboration. For the experimental version, we incorporated additional information on the rates of regret after surgical treatment found in the literature (Fernandes-Taylor & Bloom 2011; Lantz et al. 2005). In a study of 449 women 5 years post surgery for breast cancer, Fernandes-Taylor and Bloom (Fernandes-Taylor & Bloom 2011) found the post-surgical regret rate for mastectomy was 24.1 %. Lantz and colleagues (Lantz et al. 2005) reported a post-surgical regret rate of 11.4 % among a sample of 1633 women that underwent mastectomy as opposed to lumpectomy.

### Data collection and analysis

All data collection occurred in the Wharton Behavioral Lab (WBL) on the University of Pennsylvania campus. The WBL was initially funded by the Wharton School in Spring 2005. The primary goal of the WBL is to enhance the research productivity of Wharton-affiliated faculty by minimizing the operational costs, both time and money, of conducting research. Each WBL session lasts 30 to 60 min. During a session, study subjects may complete questionnaires, participate in online experiments, or interact in groups.

For the present study, standard and regret rate-incorporated decision aids for breast cancer were distributed to participants at random. All participants were asked to complete an investigator-designed computer-based survey about their treatment preferences for breast cancer surgery, as presented in the decision aid. Participants were asked to choose between lumpectomy and mastectomy and were then questioned about the role that anticipated regret played in this decision-making process and which sections or statements in the decision aid most influenced their choice for treatment.

Demographic data was collected via an online survey platform that captured age, gender, and university affiliation. Descriptive statistics were calculated for demographic data, and the anticipated regret outcomes were compared between the experimental and control groups using the chi-square test. Frequency counts were performed for each line of each decision aid and analyzed. Referenced lines from the decision aids that used the same words with different sentence structure were collapsed and counted together.

## Results

There were 189 subjects enrolled in this study. From this sample, the data collected from five of the participants were discarded due to procedural error in data collection. Of the 184 females receiving the breast cancer decision aid, 96 received the control standard version and 88 received the experimental anticipated regret-incorporated version.

Descriptive statistics of 182 study participants are available; two participants did not complete the demographic questions (Table 1). Participants were 22.0 years old on average and included undergraduates (86 %), graduate students (9 %), university staff (3 %), and others (2 %). The majority were unemployed (53.3 %), while over a third worked part-time (39.6 %) and 7.1 % worked full-time. There was a statistically significant difference between the experimental and control groups in terms of citizenship.

**Table 1:** Demographics of study subjects

	All subjects (*N* = 182)	Experimental (*N* = 88)	Control (*N* = 94)	*p* value
	Mean (SD) or *N* (%)			
Age in years	22.0 (6.6)	21.8 (5.9)	22.1 (7.2)	0.76
Employment status				
Full-time	13 (7.1)	4 (4.5)	9 (9.6)	0.43
Part-time	72 (39.6)	35 (39.8)	37 (39.3)	
Unemployed	97 (53.3)	49 (55.7)	48 (51.1)	
Academic affiliation				
Undergraduate	153 (84.1)	72 (81.8)	81 (86.2)	0.70
Graduate	21 (11.5)	13 (14.8)	8 (8.5)	
Staff	8 (4.4)	3 (3.4)	5 (5.3)	
Ethnicity				
White	54 (29.7)	24 (27.3)	30 (31.9)	0.70
Black	32 (17.6)	13 (14.8)	19 (20.2)	
Asian/Pacific Islander	68 (37.3)	35 (39.8)	33 (35.1)	
Hispanic	12 (6.6)	7 (8.0)	5 (5.3)	
Declined to state	16 (8.8)	9 (10.2)	7 (7.5)	
Citizenship				
American citizen	155 (85.1)	69 (78.4)	86 (91.5)	0.02
Green card	20 (11.0)	13 (14.8)	7 (7.4)	
No green card	2 (1.1)	1 (1.1)	1 (1.1)	
Declined to state	5 (2.8)	5 (5.7)	0	

### Quantitative data

The first outcome of interest was the difference in surgical treatment preference by the decision aid type (experimental vs. control). In the control group, which received the standard version of the decision aid, 74 out of 96 (77 %) subjects chose lumpectomy over mastectomy, whereas in the experimental group that received the regret-incorporated decision aid, 80 out of 88 (91 %) subjects chose lumpectomy over mastectomy (*p* = 0.011). Subjects in the experimental group had a threefold increased odds of choosing the less regret-inducing surgery (OR = 2.97; 95 % CI = 1.25, 7.09; *p* value = 0.014; Table 2).

**Table 2:** Preferred surgical treatment and role of regret by decision aid received

	Experimental (*N* = 88)	Control (*N* = 96)	*p* value
Treatment choice?			
Lumpectomy (vs. mastectomy)	80 (91 %)	74 (77 %)	0.011
Did regret play a role in your decision-making process?			
Yes (vs. no)	69 (78 %)	62 (65 %)	0.039
Odds of choosing lumpectomy (vs. mastectomy)			
	OR	95 % CI	*p* value
Experimental group	2.97	1.24–7.09	0.014
Odds that regret was considered in decision			
	OR	95 % CI	*p* value
Experimental group	1.99	1.03–3.84	0.04

Our other outcome of interest was whether there was a difference in consideration of regret during the decision-making process between those who received the experimental vs. the standard version of the decision aid. In the experimental group, 69 out of 88 (78 %) reported that regret was considered during the decision-making process, compared to 62 out of 96 (65 %) in the control group (*p* = 0.039). Subjects in the experimental group had a twofold increased odds (OR = 1.99; 95 % CI = 1.03, 3.84; *p* value = 0.04) of considering regret during the decision-making process as compared to subjects in the control group.

### Qualitative data

In order to analyze the responses to the survey question of “what part(s) of the brochure played a role in the decision you made for your treatment choice,” we performed frequency counts for the various sentences reported. The top five most frequent sentences for each decision aid (experimental vs. control) are presented in Table 3. Whereas many of the top five sentences reported by the control group were also among the top five most often reported by the experimental group, there was a difference in the order of importance: sentences referencing regret displaced other sentences from the control condition. This pattern demonstrated that when participants are presented with information about regret rates, this information becomes an important factor in their decision-making process.

**Table 3:** Qualitative data: top five most frequent responses by decision aid

Control group standard decision aid		Experimental group regret-incorporated decision aid	
Frequency	Response	Frequency	Response
18 (19 %)	With mastectomy, after 10 years, about 8 out of 100 women will have local recurrence. With lumpectomy and radiation, after 10 years, about 10 out of 100 women will have local recurrence	19 (22 %)	With mastectomy, after 10 years, about 8 out of 100 women who have will have local recurrence. With lumpectomy and radiation, after 10 years, about 10 out of 100 women would have a local recurrence
16 (17 %)	Mastectomy removes the entire breast	18 (20 %)	If a woman undergoes mastectomy and is unable to cope with the loss of her breast…she may regret her decision to treat a cancer that could also have been cured with breast-conserving therapy. This regret may be especially pertinent if she learns that a similar woman with a similar cancer chose lumpectomy with radiation and continued to live cancer-free without the same sacrifice to her appearance
10 (10 %)	The chance of local recurrence is low after mastectomy and slightly higher after lumpectomy with radiation	17 (19 %)	Lumpectomy saves the breast
9 (9 %)	You will live the same length of time whether you choose mastectomy or lumpectomy with radiation	14 (16 %)	24.1 % of women choosing mastectomy have regretted their decision afterwards
9 (9 %)	Cancer that comes back in the breast after lumpectomy can usually be successfully treated with mastectomy	14 (16 %)	You will live the same length of time whether you choose mastectomy or lumpectomy

## Discussion

In this randomized experimental study, we demonstrated that incorporating information about post-surgical regret into decision aids for breast cancer was associated with preferences toward a less regret-inducing surgical treatment option and a higher probability of considering regret during the decision-making process. These findings suggest that there is the potential that activating subjects to think pre-operatively about the states they may ruminate on post-operatively could inform the decision-making process. The concept that anticipated regret can be used to optimize decision-making behavior has been given credibility by recent neurobiological studies and neuroimaging data that have found a neural basis for the emotion of regret (Camille et al. 2004; Coricelli et al. 2005). Functional magnetic resonance imaging (fMRI) data have confirmed that the emotion of regret is subserved by specific cerebral regions and have localized processing to the orbitofrontal cortex (Chandrasekhar et al. 2008). The same pattern of activity expressed during the experience of regret has been demonstrated to be expressed in the moments preceding decision-making, suggesting that experienced regret and anticipated regret are mediated by the same neural circuitry (Coricelli et al. 2005). Furthermore, these same brain structures become active when learning of another individual’s regretful outcome, suggesting that one is able to incorporate this type of information into the decision-making process as it reactivates the same neural regret network (Canessa et al. 2011). Taken together, these neuroimaging studies provide a neurobiological basis for the design of our experiment and place regret at the intersection of emotion and cognition and document its role in decision behavior.

Currently, the rates of post-surgical regret and associated morbidities represent an objective body of information that has been previously overlooked or, at best, only sporadically employed by individual physicians. The observation that patients continue to have post-surgical regret rates as high as 47 % in areas of preference-sensitive medicine suggests that there is room for improvement in the way in which we currently help patients to navigate the decision-making process (Sheehan et al. 2007). Incorporating anticipated regret into the process may be one way of achieving this outcome. Further, it holds promise for combatting one of the themes most often expressed by patients experiencing regret: dissatisfaction with information provided regarding treatment alternatives and adverse side effects, which can cause patients to later say “if only I had known….” Therefore, the patient may more appropriately choose wisely from the perspective of satisfaction after the intervention.

In our experimental, hypothetical, surgical decision-making situation, receipt of the decision aid that keyed in on anticipated regret was associated with the surgical treatment option demonstrated to be less likely to induce regret. The difference in preference between the experimental and control groups is important given that studies have suggested an association between post-surgical regret and poorer health-related quality of life (Hu et al. 2003). Of note, post-surgical regret does not appear to be dependent on having a bad outcome, as it has been shown to occur in patients that are equivalent in clinical outcome measures (Schroeck et al. 2008). If anticipated regret can help patients minimize the likelihood of post-surgical regret, then it may also spare them the decrease in quality of life with which it is associated.

Despite the implications of this study, there are several limitations. Our results are limited by the hypothetical nature for the population in which the study was tested. Specifically, the study subjects were not personally facing the need to make a decision about breast cancer surgery. Further, the average age of participants in this study was younger than the age at which breast cancers are most often diagnosed (Hayat et al. 2007). Finally, our study cohort was comprised of individuals with an education beyond high school. Over 10 % were in a graduate degree program, the remainder is in an undergraduate degree program or on staff at the institution. Therefore, although this design was useful in demonstrating feasibility, the results are not immediately generalizable to the intended surgical patient population with early-stage breast cancer, a population with a more diverse educational level.

Despite these limitations, the results of this pilot study make a strong argument for applying this design to a larger, relevant surgical patient population to further investigate the role of anticipated regret in shared medical decision-making. Specifically, this study design should be repeated in cancer patients at the time of diagnosis of early-stage breast cancer. After randomization to receive the standard or regret-incorporated versions of the decision aid, patients should be followed through their decision-making process, treatment course, and post-decisional state. Important results will include the surgical treatment selected as well as the rates of post-decisional regret. We believe that a decision process which incorporates anticipated regret could be accomplished in the surgeon’s office through collaboration between the surgeon, anesthesiologist and primary caregiver as part of the consent process.

The incorporation of shared decision-making strategies into health care is becoming increasingly important with the passing of the ACA, especially in light of Section 3506, which specifically encourages their use and application. This section of the ACA establishes shared decision-making as an ideal means of accommodating patient preferences and assuring that care-delivered matches care-desired. Nevertheless, this ideal is yet to be consistently reached with less than 10 % of medical decisions meeting the standard for informed decision-making, (Braddock et al. 1999) and little being done to promote shared decision-making since the passing of the ACA. Assessing the success of medical care is a hallmark of the ACA and should include the assessment of patient-reported outcomes. This includes both health-related quality of life as well as satisfaction with care. We believe that the quality of consent should be assessed and that incorporation of anticipated regret will lead to increased satisfaction and trust.

## Conclusions

Because patient decision aids are yet to be widely implemented, we are uniquely poised to affect the future of the shared decision-making process. The results of this pilot study suggest that incorporation of regret rates into decision aids might be a potential strategy to encourage patients to think more elaborately before making a choice. Anticipated regret may be an important element of physician-patient decision-making capable of improving the quality of medical decisions and, in turn, the value of health care delivered. Our results make a strong argument for applying this design to a larger, relevant population to investigate the validity of these findings and their potential for improving shared decision-making strategies.

## Additional files

Additional file 1: Treatment Choices for Breast Cancer. Additional file 2: Treatment Choices for Breast Cancer.

## Abbreviations

ACA: Affordable Care Act
IPDAS: International Patient Decision Aids Standards
WBL: Wharton Behavioral Lab

## References

[CR1] Barry MJ, Edgman-Levitan S (2012). Shared decision making—pinnacle of patient-centered care. N Engl J Med.

[CR2] Braddock CH, Edwards KA, Hasenberg NM, Laidley TL, Levinson W (1999). Informed decision making in outpatient practice: time to get back to basics. JAMA.

[CR3] Camille N, Coricelli G, Sallet J, Pradat-Diehl P, Duhamel JR, Sirigu A (2004). The involvement of the orbitofrontal cortex in the experience of regret. Science.

[CR4] Canessa N, Motterlini M, Alemanno F, Perani D, Cappa SF (2011). Learning from other people’s experience: a neuroimaging study of decisional interactive-learning. Neuroimage.

[CR5] Chandrasekhar PV, Capra CM, Moore S, Noussair C, Berns GS (2008). Neurobiological regret and rejoice functions for aversive outcomes. Neuroimage.

[CR6] Coricelli G, Critchley HD, Joffily M, O’Doherty JP, Sirigu A, Dolan RJ (2005). Regret and its avoidance: a neuroimaging study of choice behavior. Nat Neurosci.

[CR7] Fernandes-Taylor S, Bloom JR (2011). Post-treatment regret among young breast cancer survivors. Psychooncology.

[CR8] Goel V, Sawka CA, Thiel EC, Gort EH, O’Connor AM (2001). Randomized trial of a patient decision aid for choice of surgical treatment for breast cancer. Med Decis Making.

[CR9] Hayat MJ, Howlader N, Reichman ME, Edwards BK (2007). Cancer statistics, trends, and multiple primary cancer analyses from the Surveillance, Epidemiology, and End Results (SEER) Program. Oncologist.

[CR10] Hu JC, Kwan L, Saigal CS, Litwin MS (2003). Regret in men treated for localized prostate cancer. J Urol.

[CR11] Kamenova K, Reshef A, Caulfield T (2014). Angelina Jolie’s faulty gene: newspaper coverage of a celebrity’s preventive bilateral mastectomy in Canada, the United States, and the United Kingdom. Genet Med.

[CR12] Lantz PM, Janz NK, Fagerlin A (2005). Satisfaction with surgery outcomes and the decision process in a population-based sample of women with breast cancer. Health Serv Res.

[CR13] Lewin T. Nancy Reagan defends her decision to have mastectomy. N Y Times. 1988 March;5.

[CR14] Nattinger AB, Hoffmann RG, Howell-Pelz A, Goodwin JS (1998). Effect of Nancy Reagan’s mastectomy on choice of surgery for breast cancer by US women. JAMA.

[CR15] Nelissen RM, de Vet E, Zeelenberg M (2011). Anticipated emotions and effort allocation in weight goal striving. Br J Health Psychol.

[CR16] O’Connor AM, Bennett CL, Stacey D (2009). Decision aids for people facing health treatment or screening decisions. Cochrane Database Syst Rev.

[CR17] Oshima Lee E, Emanuel EJ (2013). Shared decision making to improve care and reduce costs. N Engl J Med.

[CR18] Reyna VF, Nelson WL, Han PK, Dieckmann NF (2009). How numeracy influences risk comprehension and medical decision making. Psychol Bull.

[CR19] Schroeck FR, Krupski TL, Sun L (2008). Satisfaction and regret after open retropubic or robot-assisted laparoscopic radical prostatectomy. Eur Urol.

[CR20] Sheehan JSK, Lam T, Boyages J (2007). Association of information satisfaction, psychological distress and monitoring coping style with post-decision regret following breast reconstruction. Psychooncology.

[CR21] Simonson I (1992). The influence of anticipating regret and responsibility on purchase decisions. J Consum Res.

[CR22] Stacey D, Bennett CL, Barry MJ (2011). Decision aids for people facing health treatment or screening decisions. Cochrane Database Syst Rev.

[CR23] Wennberg JE (2002). Unwarranted variations in healthcare delivery: implications for academic medical centres. BMJ.

[CR24] Wennberg JE, Fisher ES, Skinner JS (2002). Geography and the debate over Medicare reform. Health Aff (Milwood).

[CR25] Wennberg DE, Marr A, Lang L, O’Malley S, Bennett G (2010). A randomized trial of a telephone care-management strategy. N Engl J Med.

[CR26] Zeelenberg M (1999). Anticipated regret, expected feedback and behavioral decision-making. J Behav Decis Mak.

