# Supplementary material for: Anticipated regret in shared decision-making: a randomized experimental study
Source: Perioper Med (Lond). 2016 Mar 2;5:5. doi: 10.1186/s13741-016-0031-6 (PMC4776353; doi:10.1186/s13741-016-0031-6)
Supplement: Additional file 1: — Treatment Choices for Breast Cancer. [file 13741_2016_31_MOESM1_ESM.pdf]

IN TEN YEARS:

With mastectomy, after 10 years, about 8 out of 100 women who have will have local recurrence. That means that about 92 out of 100 women will not.

With lumpectomy and radiation, after 10 years, about 10 out of 100 women would have a local recurrence. That means that about 90 out of 100 women will not.

It's important to understand that a few of the cancers that come back would have returned no matter which type of surgery was done.

MAKING YOUR DECISION:

There is no right or wrong choice of breast cancer surgery. There is only the choice that best matches your values, preferences, and way of living.

There are pros and cons with each choice, and each involves some things that you can't control. You have to be comfortable with the possible benefits
and harms of the treatment you choose.

In some medical situations there is a clear right answer, and your doctor can tell you exactly what's best to do. In other situations, like with breast cancer, there are different choices that are reasonable.

What's "best" depends on how you feel about the good and bad things that might happen with each choice.

Work with your doctor and your family to make your healthcare decisions.

With Early Breast Cancer, you have time, you have values, and you have choices.

Talk to your doctor about your various options.

YOUR DOCTOR TOLD YOU THAT YOU HAVE BEEN DIAGNOSED WITH BREAST CANCER, NOW YOU NEED TO KNOW...

TREATMENT CHOICES FOR BREAST CANCER

WHAT IS BREAST CANCER?

Normally, cells in the body grow, divide, and die in an orderly way. Sometimes, however, certain breast cells can become abnormal and grow out of control. Breast cancer is a disease in which breast cells grow out of control and form a tumor.

I HAVE EARLY BREAST CANCER; WHAT DOES THAT MEAN FOR ME?

It means that you are going to have to undergo surgery. The type of surgery you have, however, is up to you and you are going to need to make a decision with your doctor and your family.

WHAT ARE MY CHOICES?

There are several treatment options that a woman in your situation can consider. You will want to understand and discuss with your doctor and family how each treatment might affect your life. Your choices include:

* Mastectomy: Surgery to remove the breast; may or may not be followed by breast reconstruction, and possibly radiation.
* Lumpectomy: Surgery to remove the tumor with a border of health tissue around it (while sparing the breast), followed by radiation therapy to kill any cancer cells that may be left in the breast area.

What should I do?

Whether your cancer spreads quickly, slowly, or not at all does not change your surgery choices. Mastectomy and lumpectomy with radiation are equally good treatments for most early-stage invasive breast cancers. Having one of the other makes NO difference in how long you will live.

The chances of surviving breast cancer are exactly the same whether you have a mastectomy or whether you have a lumpectomy and radiation. Therefore, your own preference for those treatments becomes very important in making the decision.

THE SURGERIES:

Option 1. mastectomy:

Mastectomy is done under general anesthesia. The surgeon typically uses one cut in the skin to remove the entire affected breast and some lymph nodes under the arm. The mastectomy surgery (without reconstruction) usually takes several hours.

Following Mastectomy, some women have breast pain, premenstrual-type breast soreness, or other sensations as if the breast were still there. In about 15% of women, the pain or sensations may persist for years.

OPTION 2. Lumpectomy & radiation (breast-conserving surgery):

Lumpectomy may be done either in a hospital operating room or in an outpatient surgery center. General or local anesthesia can be used. The surgeon makes an incision in the breast and removes the tumor along with a border of healthy tissue all around it to act as a "safety zone" that helps reduce the chance that any cancer cells are left behind.

radiation therapy:

The x-ray energy from radiation therapy kills cancer cells that may be left in the breast. Radiation therapy reduces the chance of cancer coming back in the breast and breast area. Radiation therapy usually starts 4 to 6 weeks after lumpectomy, allowing the breast time to heal. Women generally have outpatient radiation treatments 5 days a week for about 6 weeks.

What to Expect with Traditional Radiation Therapy:

The radiation treatments each last about 5 to 15 minutes and are painless. Many women say radiation therapy is a lot like having a routine x-ray. Most women continue with their usual daily activities immediately after treatment.

Possible Side Effects of Radiation Therapy:

Many women have no discomfort or side effects from radiation therapy. Other women have temporary side effects that may include:

- Fatigue; Skin reactions, such as a sunburn-like rash; A sense of heaviness in the breast; Loss of appetite or nausea (less common with modern radiation techniques than with older ones)

Comparing the choices:

Women who have lumpectomy with radiation live as long as women who have mastectomy. Because there is little medical evidence to favor either option, the best decision is the one that matches your own values.

HOW THE SURGERIES ARE SIMILAR:

You will live the same length of time whether you choose mastectomy or lumpectomy with radiation. The surgeries are also similar in terms of how much discomfort you may feel, the chance of complications from the surgery itself, and the emotional effects.

How the Surgeries Are Different

Mastectomy and lumpectomy with radiation are different in three important ways:

- How your breast looks after your surgery—your appearance.
- How much time and energy your treatment involves and how much it disrupts your life.
- The chance that your cancer might come back in the breast or breast area (local recurrence).

How you feel about each of these differences will help determine which treatment is right for you.

Appearance:

Mastectomy

Mastectomy removes the entire breast. Your appearance afterward will depend on whether you choose to have breast reconstruction If you do not have reconstruction, your chest wall will be flat, with a scar.

Lumpectomy

Lumpectomy saves the breast. Your appearance—how the breast looks afterward—will depend on how much breast tissue was removed. In most cases, the breast will look fairly normal. Radiation can cause some changes in the skin of the breast or make the breast feel hard.

Local Recurrence:

The chance of local recurrence—cancer that comes back in the breast or breast area after surgery—is low after mastectomy and slightly higher after lumpectomy with radiation. Cancer that comes back in the breast after lumpectomy can usually be successfully treated with a mastectomy. Cancer that comes back after mastectomy is sometimes difficult to treat; about half of these recurrences can be treated successfully.
